# Influence of Nutrition and Physical Activity on Local and Systemic Inflammatory Signs in Experimentally Induced Gingivitis

**DOI:** 10.3390/nu15153344

**Published:** 2023-07-27

**Authors:** Ingmar Staufenbiel, Knut Adam, Andreas Hahn, Felix Kerlikowsky, Marco Flohr, Nadine Schlueter, Kirstin Vach

**Affiliations:** 1 Hannover Medical School, Department of Conservative Dentistry, Periodontology and Preventive Dentistry, Carl-Neuberg-Str. 1, 30625 Hannover, Germany; adam.knut@mh-hannover.de (K.A.); flohr.marco@mh-hannover.de (M.F.); schlueter.nadine@mh-hannover.de (N.S.); vach.kirstin@mh-hannover.de (K.V.); 2 Institute of Food Science and Human Nutrition, Am Kleinen Felde 30, 30167 Hannover, Germany; hahn@nutrition.uni-hannover.de (A.H.); kerlikowsky@nutrition.uni-hannover.de (F.K.); 3 Center for Dental Medicine, Department of Operative Dentistry and Periodontology, Faculty of Medicine and Medical Center, University of Freiburg, Hugstetter Straße 55, 79106 Freiburg, Germany

**Keywords:** nutrition, gingivitis, dietary inflammatory index, physical activity, lifestyle-associated risk factors

## Abstract

Although numerous studies have been published investigating the relationship between various dietary components and inflammatory periodontal disease, it has not yet been possible to clearly distinguish between periodontally healthy and unhealthy diets. This clinical study aimed to assess the association of specific food ingredients and physical activity on local and systemic inflammatory signs in experimentally induced gingivitis. Thirty-nine non-smoking periodontally healthy volunteers (mean age 23.2 ± 3.8 years) refrained from oral hygiene in the right maxilla for 21 days to induce an experimental gingivitis. Clinical examination (baseline and day 21) included plaque index, bleeding on probing (BOP), gingival crevicular fluid volume and high sensitive C-reactive protein levels (blood sample). Accompanying the intervention, volunteers documented with validated questionnaires their physical activity converted into metabolic equivalent (MET) and their nutrition converted into the dietary inflammatory index (DII). Significantly lower BOP (*p* = 0.039) was found for subjects with a more anti-inflammatory DII than for those with a more pro-inflammatory DII; higher MET values were correlated with lower BOP at day 21 (correlation coefficient −0.36). The results show an influence of nutrition and physical activity on periodontal inflammation signs. The DII may be a suitable parameter to verify the relationship between nutrition and inflammatory periodontal diseases.

## 1. Introduction

Inflammatory periodontal diseases are highly prevalent with 1.1 billion cases of severe periodontitis worldwide [1]. The classification of periodontal and peri-implant diseases and conditions differentiates cases of periodontal health, gingivitis and periodontitis [2]. Although gingival inflammation is regarded as the most important etiological factor in the development of periodontitis [3], there are many cases of gingivitis in which tissue homeostasis is preserved for years without developing attachment loss. In the last decade, the etiological understanding of periodontitis changed substantially, with the immuno-inflammatory host response coming more into focus. According to the current model of the pathogenesis of inflammatory periodontal diseases, periodontitis occurs when a dysbiotic biofilm, a failed resolution and a disproportionate host response come together [3]. Consequently, periodontal risk factors affecting the immuno-inflammatory host response play a key role in the pathogenesis of periodontitis. In addition to established periodontal risk factors like smoking and poorly controlled diabetes mellitus, a number of other lifestyle-associated risk factors such as physical inactivity and an unbalanced diet are playing an increasing role in periodontology [4] and also for cardiovascular disease [5,6].

Insufficient physical activity is a global problem with a prevalence of more than 40% in Germany [7] and belongs to the main causes of most non-communicable diseases (NCDs) in industrialized nations [8]. Furman et al. [9] introduced a model illustrating the importance of low-grade systemic chronic inflammation (SCI). The illustration shows the most common triggers of SCI on one side and the consequences of SCI on the other side linked by SCI presented in the center. The most common triggers of SCI include a variety of lifestyle-associated factors such as stress, obesity, etc., as well as an unbalanced diet and physical inactivity. The resulting chronic increase in the systemic inflammatory markers can lead to common diseases of the industrialized nations including metabolic syndrome, cardiovascular disease, cancer, etc. The question arises whether periodontitis is a trigger and/or a consequence of SCI. Numerous studies have shown that particularly severe forms of periodontitis can lead to systemic inflammation and that periodontal therapy can reduce systemic inflammatory markers [10]. Similarly, physical inactivity can contribute to systemic inflammation and, vice versa, exercise training can lead to a decrease of SCI [11]. Regarding nutrition, it is well known which dietary components can increase or decrease SCI [12]. However, there are several difficulties in nutritional research. Most study results are based on dietary protocols including high response bias. Additionally, nutritional science should not be one-dimensional by considering only one dietary component. Numerous studies investigated the association between specific food ingredients such as lettuce juice consumption [13] or bilberries [14] with periodontal inflammation signs without evaluating the basic diet. As a consequence, “we do not know whether dietary counselling may have a positive impact in periodontitis therapy”, which was also the answer in the EFP S3 level clinical practice guideline for the treatment of stage I-III periodontitis [4] to the question “What is the efficacy of dietary counselling in periodontitis therapy”. This lack of evidence may be a result of frequently one-dimensional nutritional research in the periodontal field.

For enhancing the evidence, not only the impacts of single dietary components but of the complete nutritional status and the physical activity were investigated in the present study. For this purpose, from nutrition protocols, the dietary inflammatory index (DII) as suggested by Shivappa et al. [15] and the weekly metabolic equivalent (MET) were calculated. The DII is based on forty-five pro- and anti-inflammatory food parameters. Consequently, most of the summation effects can be considered.

The present study was based on the following questions:

(1) Can specific food components be identified that lead to more or fewer inflammation signs in the setting of an experimentally induced gingivitis?
(2) Does an anti-inflammatory diet, defined by DII, result in lower signs of periodontal inflammation compared to a pro-inflammatory diet?
(3) Are there differences between physically active and inactive volunteers in terms of periodontal signs of inflammation?
(4) Are there synergistic effects between physical activity and anti-inflammatory diet?

## 2. Materials and Methods

### 2.1. Volunteers

Volunteers were recruited via a public announcement in Hannover Medical School. Fifty-two voluntary subjects were screened at the Department of Conservative Dentistry, Periodontology and Preventive Dentistry and selected according to the following inclusion criteria: 20–30 years of age, non-smokers, no clinical signs of gingival inflammation (redness, swelling, bleeding), no probing pocket depth more than 3 mm at any site and no alveolar bone loss. Exclusion criteria were as follows: presence of systemic diseases (e.g., diabetes mellitus or cardiovascular, kidney, liver or lung disease), pregnancy or breastfeeding, history of drug abuse, allergic diathesis, medications—in particular ingestion of non-steroidal or steroidal anti-inflammatory drugs—analgesics or antibiotics within the last 3 months, implants, teeth with untreated carious lesions and/or insufficient direct or indirect restorations in the right maxilla and mouth breathing. Thirty-nine subjects met the inclusion criteria.

Detailed instructions were given to the volunteers including an information brochure explaining the study design. All subjects signed an informed consent form.

### 2.2. Study Design

Fourteen days prior to the start of the study all volunteers underwent a dental and periodontal screening in order to ensure compliance with the inclusion criteria. For all volunteers, a professional scaling and polishing of all tooth surfaces was conducted to remove supra- and subgingival plaque, staining and calculus. The intervention was initiated at baseline with oral hygiene cessation. The volunteers were instructed to refrain from any oral hygiene procedures at seven teeth of the right upper jaw over a period of 21 days. Additionally, the use of chewing gum and mouth rinse was forbidden—only local cleaning of the remaining teeth was permitted. At day 21, the experimental gingivitis phase ended, and a professional tooth cleaning and topical fluoride application was conducted. Afterwards, the volunteers resumed oral hygiene procedures.

### 2.3. Blood Samples

Fasting serum and plasma samples were obtained at baseline and at day 21 in the morning between 7.45 and 8.30 a.m. Serum concentrations of high sensitive C-reactive protein levels (hs-CRP) were determined with immunonephelometry (detection limit 0.23 mg/L, Cardiophase hsCRP, Siemens, Munich, Germany) [16].

### 2.4. Clinical Examination

The clinical examination was performed by three experienced and calibrated dentists at baseline and day 21. Clinical examination included a quantitative assessment of dental plaque and gingival inflammation. The three examiners were calibrated on five volunteers who were not included in the study.

Dental plaque was assessed visually without staining at the buccal and oral surfaces of the seven teeth of the right maxilla using a modification of the Silness–Löe plaque index (PI) [17].

For quantitative assessment of the gingival inflammation, the following parameters were determined: gingiva index (GI) [18], gingival crevicular fluid volume (GCF) and bleeding on probing (BOP). The GCF was collected with a paper strip (Periopaper, Pro Flow Incorporated, Amityville, NY, USA) after gentle drying of the tooth for 10 s. The strip was inserted for 30 s into the gingival sulcus at four sites of the upper right first premolar (mesio- and disto-buccal as well as mesio- and disto-oral). GCF was measured with a calibrated Periotron 6000 gingival fluid meter (Pro Flow Incorporated) and expressed in Periotron units (PU). Afterwards, the BOP was recorded at four sites of the seven teeth in the right maxilla (mesio- and disto-buccal, mesio- and disto-oral). For probing, a pressure-calibrated probe (TPS probe, Vivacare, Vivadent, Schaan, Liechtenstein) was used. The BOP was calculated by dividing the total number of positive scores by the total number of probed surfaces and was expressed in percent.

### 2.5. Documentation and Analysis of Physical Activity and Nutrition

Accompanying the experimental gingivitis phase, the volunteers documented their individual physical activity and nutrition by using the documentation sheet of the German Society of Nutritional Research (Gesellschaft für Ernährungsforschung e.V.).

Regarding the individual physical activity, the volunteers documented the kind, the frequency and the duration of sports. Based on these three parameters and according to the compendium of physical activities [19], the weekly metabolic equivalent (MET) was calculated and expressed in MET hours per week (METh/w) for each volunteer.

The individual consumption of food and drinks was documented daily by each volunteer. The analysis of the documentation sheet was performed in the School of Dieticians, Hannover Medical School, by using the nutrition consulting software Prodi Version 6.0 expert (Nutri-Science GmbH, Freiburg, Germany). Output of this software included the amount of daily consumption of numerous amino acids, carbohydrates, fatty acids, mineral nutrients, vitamins and large calorie.

### 2.6. Calculation of the Dietary Inflammatory Index (DII)

The DII is an established food score that reflects the inflammatory potential of individual diets. Its development is described in detail by Shivappa et al. [15]. In brief, the DII was developed to find associations between 45 food patterns and commonly used biomarkers of inflammation (C-reactive protein (CRP), IL-1β, IL-4, IL-6, IL-10, TNF-α). Depending on the weighted number of publications and study design, each food in the DII is characterized as pro- or anti-inflammatory with a raw inflammatory effect score. In addition, 11 global food consumption datasets were used to calculate a global mean daily intake of each food in the DII. This allows the raw inflammatory effect score to be standardized in relation to the global mean daily intake to obtain the total inflammatory effect scores. Within the DII, a more negative score represents an anti-inflammatory score, whereas a more positive score indicates a pro-inflammatory potential of the diet. In this study, we assessed the dietary intake using 3-day food records. Based on these data, we used 25 food items available (listed in the Appendix A) to calculate the DII.

Specifically, we first Z-scaled the calculated amount of each food item with the global mean daily intake and standard deviation to create a Z-score. The Z-score is a multiplier that reflects the intake of each individual in this study relative to the global mean.

After that, a transformation to centered percentiles was performed (first doubling and then subtracting 1) to minimize the influence of extreme values, which could otherwise bias the calculated values. Finally, the centered percentile score was multiplied by the corresponding effect score to obtain the final DII score for each food. All individual DII scores of a volunteer were then added to the total DII.

### 2.7. Statistical Analysis

A sample size calculation was made for the primary aim of the study, namely to investigate whether experimental gingivitis enhances systemic markers of inflammation [16]. With a sample size of 40, a change in the hs-CRP value of 0.25 could be detected with a power of 90% and an alpha of 5%. For our setting with 39 volunteers, a correlation of 0.5 can be detected with 80% power using a two-sided test of the null hypothesis that the correlation is 0 assuming a 5% significance level.

Mean values and standard deviations were computed for descriptive analysis. Box and scatter plots were used for graphical presentation.

Two-sided Wilcoxon signed-rank tests for paired samples were used to test for changes between baseline and day 21. As the values of the dental parameters GI, PI and BOP were close to 0 at baseline due to professional dental cleaning, further analyses were performed for the values at day 21, while, for GCF and hs-CRP, the changes (difference between day 21 and baseline) were considered. Pearson’s correlation coefficients were calculated to investigate the relationship between inflammation, on the one hand, and body mass index (BMI), individual nutritional parameters and MET, on the other. The DII was used to divide the volunteers based on the median DII value in a group with a more anti-inflammatory (DII^A^) and a group with a more pro-inflammatory (DII^P^) nutrition. To test for DII-group differences, *t*-tests for independent samples were used for GI, PI, BOP and GCF, while, due to the skewness of the distribution, a two-sided Wilcoxon rank-sum test was applied for hs-CRP. Depending on the median, the volunteers were divided into a physically active (sport+) and a physically inactive group (sport-). A linear regression with interaction of sport and DII on BOP was used to separate the single effects. For subsequent pairwise comparisons, the method of Scheffé was used to correct for multiple testing.

The significance level was set to 0.05. All computations were performed with STATA (Version 17.0, College Station, TX, USA).

## 3. Results

Thirty-nine volunteers (6 men, 33 women) with a mean age of 23.2 years (SD 3.8) were included in the present study. As expected, experimentally induced gingivitis developed in all volunteers resulting in a significant increase in the clinical inflammatory parameters GI, BOP and GCF. An overview of the clinical, laboratory and nutritional parameters is given in Table 1.

Local inflammation was followed by elevated hs-CRP values at day 21. However, this increase did not reach statistical significance. Regarding the BMI and according to the S3-guideline “Prevention and therapy of obesity” [20], the majority of volunteers were of normal weight (n = 29), two were underweight and four showed as overweight or obese. While the BMI showed a strong impact on the hs-CRP value at baseline (correlation coefficient *ρ* = 0.54, *p* = 0.001), no significant association with the change of the clinical inflammatory parameters was observed.

### 3.1. Clinical Inflammatory Parameters and Their Association to Nutrition

To investigate whether specific food ingredients were associated with a reduced or upregulated inflammatory reaction to the accumulated biofilm, a univariate analysis of single nutritional components was conducted. It revealed that a higher intake of vitamin C was significantly correlated with decreased BOP values (*ρ* = −0.40, *p* = 0.029). Furthermore, a moderate correlation between BOP and the quotient omega-6/omega-3 fatty acid (*ρ* = 0.3480, *p* = 0.060) was observed.

For the more sensitive parameter GCF, only vitamin E (*ρ* = −0.46, *p* = 0.011) showed a significant anti-inflammatory effect.

### 3.2. Association to Dietary Inflammatory Index

**Figure 1:**
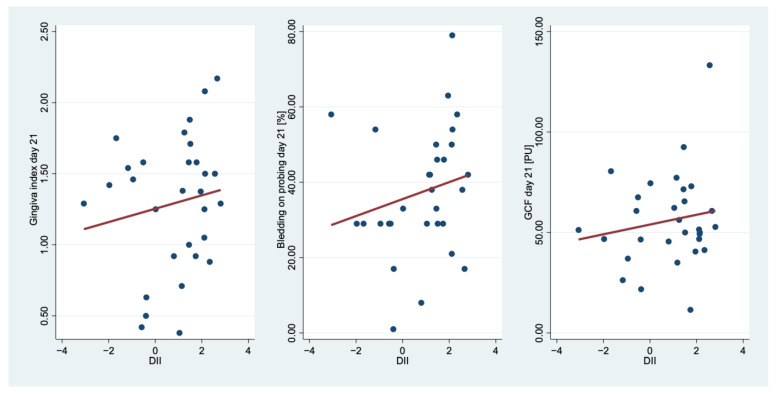
Association between the dietary inflammatory index (DII) and gingiva index (GI), bleeding on probing (BOP) and gingival crevicular fluid (GCF) at day 21. Red line: estimated linear regression line.

In order to consider the most important summation effects, the DII was calculated and its relationship to the GI, the BOP and the GCF were examined (Figure 1).

The scatter plots (Figure 1) show that the more pro-inflammatory the diet, the higher the clinical inflammatory parameters at day 21. For the BMI, we observed a negative correlation (*ρ* = −0.28, *p* = 0.143) to the DII.

**Figure 2:**
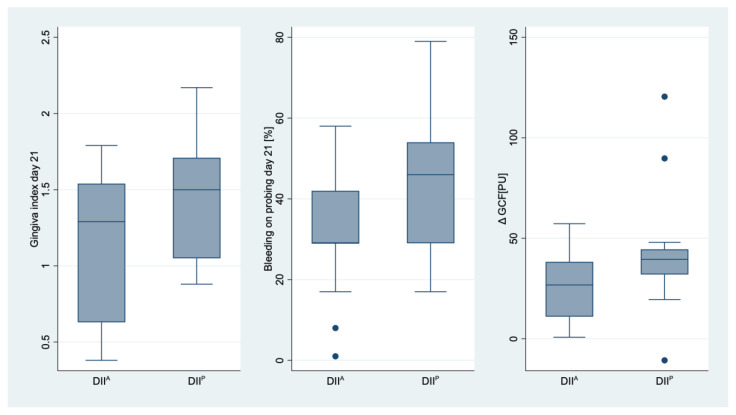
Distribution of gingiva index and bleeding on probing on day 21, and change of sulcus fluid (ΔGCF) for the group with pro- (DII^P)^ and anti-inflammatory (DII^A^) nutrition. Significant differences were only found for bleeding on probing.

Based on the median DII value (1.34), a DII^P^- and a DII^A^-group were differentiated. In the DII^A^-group, BOP at day 21 was significantly lower than in the DII^P^-group (*p* = 0.039), while no difference was found for GI (*p* = 0.064), PI (*p* = 0.167), GCF (*p* = 0.076) and hs-CRP (*p* = 0.801) (Figure 2).

### 3.3. Association to Physical Activity

Physical activity was raised for most volunteers (n = 30) and converted in MET hours per week (METh/w). The volunteers showed a wide range in the extent of their sporting activities (mean 21.5 METh/w, SD 18.2). For the BOP-value at day 21, a correlation of −0.36 (*p* = 0.054) was found. As shown for DII, METh/w were negatively correlated with BOP (*ρ* = −0.19, *p* = 0.318). For GI (*ρ* = 0.13), PI (*ρ* = 0.01), GCF (*ρ* = 0.02), hs-CRP (*ρ* = 0.01) and BMI (*ρ* = 0.03), the correlation to MET was negligible.

**Figure 3:**
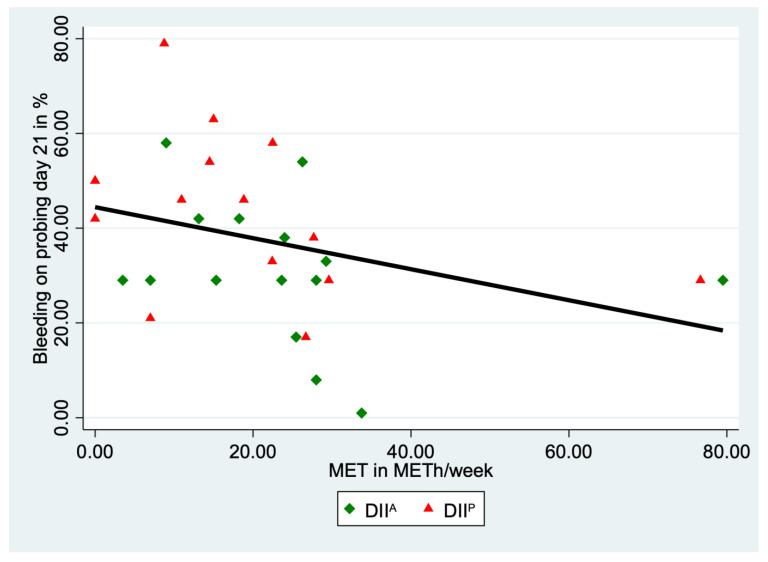
Bleeding on probing with a dependence on metabolic equivalent (MET) for volunteers of the anti-inflammatory (DII^A^, green squares) and pro-inflammatory (DII^P^, red triangles) nutrition groups. Black line indicates the fitted regression line over all volunteers.

An additional interaction analysis was carried out for DII and physical activity to investigate possible synergistic anti-inflammatory effects. We observed the lowest BOP values at day 21 in physically active volunteers consuming an anti-inflammatory diet (DII^A^ & sport^+^, BOP: 26%), followed by the DII^P^ & sport^+^-group (BOP: 34%), the DII^A^ & sport^−^-group (38%) and the DII^P^ & sport^−^-group (BOP: 50%, Figure 3). Only the difference between the groups DII^A^ & sport^+^ and DII^P^ & sport^−^ reached statistical significance (*p* = 0.020).

The scatter plot (Figure 3) shows that the more physically active the volunteers were, the lower the BOP values became at day 21, indicating that physical activity seems to have an anti-inflammatory effect. Volunteers with a more anti-inflammatory nutrition (DII^A^, shown as green squares) have lower BOP values than those with a more pro-inflammatory nutrition (DII^P^, shown as red triangles). No significant differences in physical activity between DII^A^- and DII^P^-group were observed (*p* = 0.413).

## 4. Discussion

In this study, the association of specific food ingredients and physical activity on local and systemic inflammatory signs in volunteers with experimentally induced gingivitis was investigated. As expected, experimentally induced gingivitis developed in all volunteers after 21 days of absence of oral hygiene procedures, resulting in a significant increase in all clinical inflammatory parameters (Table 1).

When the individual food components were examined, vitamin C was the only one with a significant effect on BOP at day 21. Vitamin C is one of the most effective antioxidants [21,22]. The impact of vitamin C deficiency on periodontal tissues has been described already in the 18th century. Sailors suffered from scurvy, a disease characterized by gingival bleeding and tooth mobility caused by a hypovitaminosis of vitamin C [23]. Moreover, Chapple et al. [24] provided the first strong evidence of an inverse association between serum antioxidant concentrations and the prevalence of periodontitis in a sample of 4480 adults examined in the National Health and Nutrition Examination Survey (NHANES). According to the recent consensus report of the joint EFP/ORCA workshop, there is evidence from several studies revealing an inverse association between intake and plasma levels of vitamin C, on the one hand, and the prevalence of periodontitis, on the other hand [25]. Amaliya et al. [26] investigated the effect of vitamin C on gingival inflammatory signs in a randomized three-arm study. In an experimental gingivitis model, the daily supplementation of vitamin C (natural or synthetic) was compared with a control group. In both supplementation-groups, fewer inflammation signs were observed. Vitamin C is mainly found in fruits and vegetables. In addition, a diet rich in fruits and vegetables contains a variety of antioxidants and other phytochemicals, such as polyphenols, which are also known to have anti-inflammatory properties [27]. In a cross-sectional analysis with 10,000 adults, a high intake of fruits, vegetables, salad, poultry and seafood was associated with less clinical attachment loss [28]. Furthermore, in 1005 middle-aged Chinese women, higher consumption of cruciferous vegetables was associated with lower levels of pro-inflammatory cytokines [29]. Apart from periodontitis, epidemiological studies have shown that vegetable consumption is inversely associated with the risk of cardiovascular disease and that the protective mechanism is due to anti-inflammatory effects of vegetable intake [30].

In addition, the present study revealed a significantly inverse association between vitamin E and ∆GCF between day 21 and baseline. Vitamin E belongs to the lipid-soluble vitamins and consists of eight subgroups. Alpha-tocopherol is the most abundant form in nature and has both anti-inflammatory and anti-thrombotic effects [31]. The relationship between vitamin E intake and inflammatory periodontal disease has been widely studied, but no consistent results were obtained. In the Third US NHANES, less severe forms of periodontitis were found in participants with elevated serum alpha-tocopherol levels after adjusting for numerous confounding factors [32]. An animal study, investigating the effect of systemically administrated vitamin E and/or sodium selenite on experimentally induced periodontitis, was able to demonstrate reduced periodontitis progression and reduced inflammation signs [33]. The main sources of vitamin E are oils, nuts and green leafy vegetables. A diet rich in these foods contains phenolic compounds and omega-3 fatty acids, which have also been shown to reduce inflammation. The evidence suggests that dietary fat sources play a key role in regulating pro- and anti-inflammatory processes. Saturated fats from animal products are associated with pro-inflammatory pathways, whereas unsaturated fatty acids from oils and nuts may be causal for the anti-inflammatory effects investigated in this study, with vitamin E as a related marker for nuts and oils [34,35].

However, a healthy diet is not characterized by the supplementation of a few anti-inflammatory food ingredients or specific nutritional supplements. If pro-inflammatory food components are consumed in large amounts at the same time, the overall food balance remains pro-inflammatory. Therefore, the DII was calculated to account for the most important summation effects. After a dichotomization based on the DII, significantly higher BOP values were found at day 21 in the pro-inflammatory compared to the anti-inflammatory nutrition group. This means that by summarizing the dietary parameters into the dietary inflammatory index, we obtained a multidimensional view of the diet of the volunteers. Only through this multidimensional view were we able to demonstrate that a pro-inflammatory diet is associated with higher clinical signs of inflammation.

In a recent cross-sectional study [36] with 1570 participants, the most anti-inflammatory DII was observed with the highest intake of nuts, fruits, vegetables, seeds and low-calorie drinks. In contrast, consumption of sweetened carbonated soft drinks, refined grains and red meat was associated with a more pro-inflammatory diet. However, in the present analyses we were not able to include liquids in the calculation of the DII. Nevertheless, the calculation of the DII was based on dietary data from a very homogeneous group of predominantly young women. It can be assumed that, within young women, the consumption of pro-inflammatory sweetened drinks is about the same as the consumption of anti-inflammatory drinks such as tea and coffee.

In the current literature, there are many studies investigating the effect of specific food components on periodontal inflammatory parameters. Jockel-Schneider et al. [13] investigated the impact of dietary nitrate from repeated lettuce juice consumption on gingival inflammation. Widén et al. [14] examined the effect of bilberry consumption on gingival inflammation signs. Both studies revealed an anti-inflammatory effect of the investigated food components. However, the effect was small and the daily-consumed amounts of up to 500 mg of bilberries were not suitable for everyday use. Considering specific diets such as a Mediterranean diet or whole-food nutrition, anti-inflammatory effects in a clinically relevant range could be observed in different studies. Woelber et al. examined the effect of a multidimensional nutritional intervention with a reduced intake of processed carbohydrates and an increased intake of micronutrients. The change in nutrition resulted in significantly decreased GI values (baseline: 1.04, after 8 weeks: 0.61) in a clinically relevant range without a dental intervention [37]. Bartha et al. investigated the effect of a Mediterranean diet intervention for six weeks on gingival inflammatory parameters in a randomized controlled trial. BOP values decreased in the intervention group, again in a clinically relevant range (baseline: 51%, after 6 weeks: 39.93%) [38]. Even if the sample sizes in these studies were small (30 and 42 cases, respectively), multidimensional nutritional intervention seems to be very effective in reducing gingival inflammation signs.

Comparable data were found in a Korean population-based cohort study. The association between the inflammatory potential of diet and the risk of periodontitis was examined in 160,397 adults. The DII was used to divide the study cohort into quartiles. The subjects with the most pro-inflammatory diet showed a seventeen percent higher risk for periodontitis compared to those with the most anti-inflammatory diet [39]. Additionally, Machado et al. demonstrated in a NHANES sample of 10,178 subjects a significant association between DII and mean probing-pocket depth and mean clinical attachment level, respectively. In contrast to the present study, the underlying periodontal data lacked measures of local inflammation such as BOP [40]. In addition, most volunteers of the present study documented their physical activity during the experimental gingivitis phase. A moderate correlation [41] of −0.36 was found between the physical activity and the BOP value at day 21, suggesting that physical activity can be another dimension for reducing gingival inflammation signs. Few studies in the scientific literature have investigated the relationship between physical activity and inflammatory periodontal disease. In a blinded, randomized, controlled trial, the influence of physical activity on periodontal health was investigated. Thirty-seven patients with type 2 diabetes were assigned to an intervention (physical activity over a period of six month) or the control group. The intervention resulted in a reduction of inflammation signs (BOP decreased from14.5 to 2.4%) and probing pocket depth (decreased from 2.27 to 1.38 mm), with no significant changes in the control group [42]. In a Japanese prospective intervention study, 71 overweight, physically inactive men were subjected to either an exercise (n = 50) or a dietary intervention (n = 21) over 12 weeks. The number of teeth with a probing-pocket depth of more than 3 mm and a positive BOP significantly decreased (PPD: 14.4 to 5.6%; BOP: 39.8 to 14.4%) in the exercise group [43]. In another study, six months of training in patients with type 2 diabetes led to a significant reduction in haemoglobin A1c, the papillary bleeding score and the probing-pocket depths [35]. Regarding the association between physical activity and the prevalence and severity of periodontitis, several studies demonstrated an inverse association [44,45,46,47]. In conclusion, the evidence on the association between physical activity and inflammatory periodontal disease is small but consistent.

A subsequent interaction analysis even showed that the combination of physical activity and an anti-inflammatory diet had the greatest effect on periodontal inflammation signs, supporting the assumption that a generally healthy lifestyle has a positive effect on periodontal heath.

Despite the conclusive results, our study has some limitations. Regarding the study population, we investigated a rather small cohort of young and healthy volunteers with an unequal gender distribution. Therefore, the influence of gender could not be estimated. The choice of a homogeneous age group had the advantage that an adjustment for age effects was not necessary, but it raises the question of transferability of the results to the general population. Considering the documentation and analysis of nutrition and physical activity through documentation sheets, an increased response bias must be assumed due to deliberate misstatements especially among volunteers with an unhealthy diet and insufficient physical activity. In addition, there are some limitations of the DII. The DII provides information about the inflammatory potential of a diet based on the micronutrients and whole nutrient groups consumed and does not take into account the degree of the processing of the food and the texture of the food. For example, highly processed foods may have different effects on inflammatory processes than predominantly unprocessed food. In addition to the parameters studied, an analysis of the oral microbiome could have provided new insights, since diet and physical activity naturally interact with the oral microbiome. Whether the three weeks would have been sufficient for a decisive change in the microbiome remains questionable [48,49].

## 5. Conclusions

Returning to our initial questions, we can state that, of the specific food components, only vitamin C, vitamin E and the omega-6/omega-3 fatty acid quotient showed a correlation with inflammatory signs in the setting of an experimentally induced gingivitis. It was shown that the more pro-inflammatory the diet was, the higher the clinical inflammatory parameters were when observed at the end of the study. For physical activity alone, only a correlation with BOP at day 21 was found. Physical activity and anti-inflammatory diet seem to have synergistic effects. The DII could be a suitable measure for future studies to examine the relationship between diet and periodontal inflammatory disease in a larger, more heterogeneous population.

## Figures and Tables

**Table 1:** Mean values (± standard deviation) for clinical, laboratory and nutritional parameters at baseline and day 21. More details of the study population can be found in Eberhard et al. [16].

Parameter	Baseline	Day 21	*p*-Value
PI	0.04 (±0.11)	2.06 (±0.36)	<0.001
GI	0.06 (±0.08)	1.31(±0.44)	<0.001
BOP [%]	5.36 (±5.81)	37.97 (±15.59)	<0.001
GCF [PU]	23.41 (±17.55)	56.01 (±22.04)	<0.001
hs-CRP [mg/L]	1.43 (±2.02)	2.03 (±2.50)	0.248
BMI [kg/m^2^, n = 35]	22.90 (±4.51)		
DII (n = 30)	0.83 (±1.53)		
MET [METh/w, n = 30]	21.49 (±18.25)		

Abbreviations: PI: plaque index; GI: gingiva index; BOP: bleeding on probing; GCF: gingival crevicular fluid volume; hs-CRP: high sensitive C-reactive protein levels; BMI: body mass index; DII: dietary inflammatory index; MET: metabolic equivalent.

## Data Availability

The dataset used is available from the corresponding author on reasonable request.

## Appendix A

The following nutrition parameters were investigated and used for the DII: fiber, vitamin A, energy, B2, iron, magnesium, zinc, alcohol, cholesterol, folate, B1, B12, B6, vitamin C, vitamin D, vitamin E, carbohydrates, protein, niacin, saturated fatty acids, omega 3 and omega 6 fatty acids, monounsaturated fatty acid (MUFA) and polyunsaturated fatty acids (PUFA).
